# Immunohistochemistry as a Cornerstone in Lung Cancer Diagnosis: Subtyping Based on the 2021 World Health Organization Classification and Its Practical Application

**DOI:** 10.14740/wjon2757

**Published:** 2026-05-08

**Authors:** Imrana Tanvir, Amber Hassan, Bushra Nisar, Sadia Khan, Maryam Altaf, Humaira Waseem, Hussain Noorwali, Ali A. Mousa, Amany Fathaddin, Ragdah Arif, Mohammed M. Karami, Majid Almansouri

**Affiliations:** a Department of Pathology, Faculty of Medicine, King Abdulaziz University, Rabigh, Saudi Arabia; b European School of Molecular Medicine, University of Milan, Milan, Italy; c Ameerudeen Medical College/PGMI/LGH Lahore, Lahore, Pakistan; d BPP University, Manchester, UK; e Fatima Jinnah Medical University, Lahore, Pakistan; f Department of Basic Medical Sciences, College of Medicine, University of Jeddah, Jeddah, Saudi Arabia; g Department of Anatomy and Embryology, Faculty of Medicine, Jouf University, Jouf, Saudi Arabia; h Department of Pathology, Faculty of Medicine, King Saud University, Riyadh, Saudi Arabia; i Department of Internal Medicine, Faculty of Medicine, King Abdulaziz University, Jeddah, Saudi Arabia; j Department of Clinical Physiology, Faculty of Medicine, King Abdulaziz University, Jeddah, Saudi Arabia; k Department of Clinical Biochemistry, Faculty of Medicine, King Abdulaziz University, Jeddah, Saudi Arabia

**Keywords:** Lung carcinoma, Immunohistochemistry, Adenocarcinoma, Squamous cell carcinoma, Neuroendocrine tumor, WHO classification, Metastasis

## Abstract

**Background:**

Lung cancer is the leading cause of cancer-related deaths worldwide, and precise histological classification is vital for therapy decisions. Given diagnostic limitations with small biopsies, immunohistochemistry (IHC) enhances accuracy. This study evaluated the diagnostic value of IHC markers in subtyping lung tumors per the 2021 World Health Organization (WHO) classification and differentiating primary from metastatic lesions.

**Methods:**

A prospective study was conducted on 151 lung biopsy specimens over 18 months. All samples were processed using standard histopathological techniques and an IHC panel comprising thyroid transcription factor-1 (TTF-1), napsin A, cytokeratin (CK)7, CK5/6, P63, synaptophysin, and chromogranin. Additional markers (estrogen receptor (ER), progesterone receptor (PR), human epidermal growth factor receptor 2 (HER2), PAX-8, PAX-2, CD10) were applied for metastatic differentiation. Statistical correlation analysis between lineage-specific markers was performed to assess diagnostic concordance.

**Results:**

Adenocarcinoma was the most prevalent subtype (58.3%), followed by neuroendocrine neoplasms (17.2%), metastatic lesions (13.2%), adenosquamous carcinoma (6.0%), and squamous cell carcinoma (2.6%). Rare entities included carcinosarcoma and mucoepidermoid carcinoma (each 1.3%). TTF-1 and napsin A were highly specific for adenocarcinoma (85% and 94%, respectively), whereas CK5/6 and P63 confirmed squamous differentiation (100% each). Synaptophysin and chromogranin were positive in 96% and 100% of neuroendocrine tumors, respectively. Correlation analysis demonstrated strong marker concordance: TTF-1 vs napsin A (r = 0.88, P < 0.001), P63 vs CK5/6 (r = 0.93, P < 0.001), and synaptophysin vs chromogranin (r = 0.87, P < 0.001). All metastatic lesions were TTF-1 negative, confirming their extrapulmonary origin.

**Conclusions:**

Combining IHC with histopathology markedly improves diagnostic precision and consistency in lung tumor classification. Lineage-specific markers validate the IHC panel’s reliability, distinguishing adenocarcinoma, squamous, and neuroendocrine types while differentiating primary from metastatic lesions. This integrative approach supports WHO-aligned precision diagnostics and guides individualized therapeutic strategies.

## Introduction

Lung cancer remains the leading cause of cancer-related mortality worldwide, accounting for nearly one in five cancer deaths annually [1]. Histologically, lung tumors represent a heterogeneous group of malignancies arising from epithelial, mesenchymal, or neuroendocrine origins [2]. Among these, non-small cell lung carcinoma (NSCLC) constitutes approximately 85% of all lung cancers. It is further classified into adenocarcinoma, squamous cell carcinoma, and large cell carcinoma, each characterized by distinct morphological and molecular features [3–5].

In recent decades, adenocarcinoma has surpassed squamous cell carcinoma as the predominant histological subtype, a trend attributed to changing smoking habits, environmental exposures, and genetic susceptibility [6, 7]. The latest World Health Organization (WHO) classification of lung tumors integrates histopathological, molecular, and radiological criteria to enable accurate subclassification and to guide targeted therapeutic strategies [5, 8].

Despite advances in radiological imaging and minimally invasive diagnostic techniques, a large proportion of lung cancers are diagnosed at advanced stages when only limited biopsy or cytological material is available [9–11]. Under these circumstances, morphological evaluation alone is often insufficient, especially in poorly differentiated or overlapping histological patterns [4, 12]. Therefore, immunohistochemistry (IHC) has become an indispensable adjunct to conventional histopathology, providing lineage-specific information that refines diagnosis and informs management [13].

IHC enables accurate subtyping of NSCLC and facilitates differentiation from other tumor entities, including neuroendocrine and metastatic lesions [11, 14]. Markers such as thyroid transcription factor-1 (TTF-1) and napsin A are specific for adenocarcinoma, whereas cytokeratin (CK)5/6, P63, and its isoform P40 reliably indicate squamous differentiation [15–17]. Synaptophysin and chromogranin are essential markers of neuroendocrine differentiation, whereas CK7 expression is observed in most epithelial tumors and supports lineage determination [18].

While the diagnostic role of individual immunohistochemical markers is well established, their combined performance and concordance in routine clinical practice, particularly in small biopsy specimens, remain critically relevant. In real-world diagnostic settings, limited tissue availability often restricts morphological assessment, necessitating reliance on optimized immunohistochemical panels [10, 19]. This study therefore provides a systematic, data-driven evaluation of a focused IHC panel within the framework of the WHO 2021 classification, with emphasis on marker concordance, diagnostic reliability, and practical applicability in routine pathology workflows, including the distinction between primary and metastatic lesions [20–22].

## Materials and Methods

### Study design and duration

This prospective descriptive study was conducted in the Department of Pathology at a tertiary care teaching hospital over 18 months, from February 2023 to December 2024. A total of 151 lung biopsy specimens were included based on histopathological suspicion or radiological evidence of malignancy. Ethical approval for the study protocol was obtained from the Institutional Review Board, and the study was conducted in accordance with the Declaration of Helsinki.

### Sample selection and criteria

A total of 151 consecutive lung biopsy specimens were included, comprising both bronchoscopic and transthoracic needle core biopsies. Only cases showing malignant pulmonary lesions with adequate tissue for histopathological and IHC evaluation were enrolled. Non-neoplastic, inflammatory, or inadequate biopsies were excluded from the study. Relevant clinical and radiological data were retrieved from patient records to aid in diagnostic correlation.

### Tissue processing and histopathological examination

Biopsy samples were immediately fixed in 10% neutral buffered formalin for 6–24 h and processed through graded alcohols for dehydration, followed by xylene for clearing and paraffin embedding. Tissue blocks were sectioned at 3–5 µm thickness using a rotary microtome. Routine hematoxylin and eosin (H&E) staining was performed to assess tumor morphology and to establish a preliminary diagnosis.

### Immunohistochemical analysis

Immunohistochemical staining was carried out on formalin-fixed, paraffin-embedded (FFPE) tissue sections using the standard streptavidin–biotin peroxidase technique. Sections were deparaffinized in xylene, rehydrated through descending grades of alcohol, and subjected to antigen retrieval in citrate buffer (pH 6.0) using a microwave-based protocol. Endogenous peroxidase activity was blocked using 3% hydrogen peroxide. The primary antibody panel included TTF-1 (clone 8G7G3/1) and napsin A as markers for adenocarcinoma differentiation; CK7 as an epithelial marker for NSCLC; CK5/6 and P63 as markers for squamous cell carcinoma differentiation; and synaptophysin and chromogranin as neuroendocrine differentiation markers.

In selected cases with suspected metastasis, additional site-specific markers were used, including estrogen receptor (ER), progesterone receptor (PR), human epidermal growth factor receptor 2 (HER2), PAX-8, CD10, and E-cadherin to identify primary sites such as breast, renal, or oral cavity carcinomas [14, 19]. Immunoreactivity was visualized using diaminobenzidine (DAB) as the chromogen and counterstained with Mayer’s hematoxylin. Positive and negative controls were included with each staining batch to ensure accuracy and reproducibility. The expression pattern for each marker was evaluated semi-quantitatively based on staining intensity and the percentage of tumor cells stained.

### Diagnostic categorization

All cases were reviewed independently by two experienced histopathologists. Final diagnoses were established by integrating morphological features and immunohistochemical profiles in accordance with the 2021 WHO classification of thoracic tumors [5].

### Statistical analysis

All data were analyzed using IBM SPSS Statistics version 26.0 (IBM Corp., Armonk, NY, USA). Descriptive statistics were applied to summarize demographic and pathological variables. Categorical data, including age group, gender distribution, histopathological subtype, and IHC expression patterns, were presented as frequencies and percentages.

The diagnostic specificity of each IHC marker was determined by correlating its staining profile with the final histopathological diagnosis. Pearson’s correlation coefficient (r) was calculated to assess the strength and direction of associations between lineage-specific markers, including TTF-1 vs napsin A, P63 vs CK5/6, and synaptophysin vs chromogranin. A P value < 0.05 was considered statistically significant, while correlations with P < 0.001 were interpreted as highly significant.

## Results

### Demographic profile

A total of 151 lung tumor cases were analyzed over 18 months. Patient ages ranged from 30 to 82 years (mean ± standard deviation (SD), 61.8 ± 9.4 years). The highest incidence was observed in the 51–60-year age group (36.4%), followed by the 61–70-year age group (33.1%) and the > 70-year age group (18.5%). Patients aged 41–50 years accounted for 9.3% of cases, while those aged 30–40 years accounted for 2.7%. The study population exhibited a clear male predominance, comprising 103 males (68.2%) and 48 females (31.8%), yielding a male-to-female ratio of 2.14:1

### Primary versus metastatic lesions

Of the 151 cases, 131 (86.8%) were primary lung tumors, and 20 (13.2%) were secondary metastatic deposits. The most common sources of metastasis included the breast (10 cases), kidney (six cases), and oral cavity (four cases). All metastatic lesions were morphologically confirmed and further validated by IHC using organ-specific markers.

### Histopathological spectrum

Adenocarcinoma was the predominant subtype, observed in 88 cases (58.3%), followed by neuroendocrine neoplasms (26 cases, 17.2%), and metastatic lesions (20 cases, 13.2%). Other less frequent subtypes included adenosquamous carcinoma (nine cases, 6.0%), squamous cell carcinoma (four cases, 2.6%), and rare entities such as carcinosarcoma and mucoepidermoid carcinoma (each 1.3%) (Table 1).

**Table 1:** Distribution of Lung Tumor Subtypes (N = 151)

Histopathological diagnosis	Frequency	Percentage (%)
Adenocarcinoma	88	58.3
Lung neuroendocrine neoplasms	26	17.2
Metastatic lesions	20	13.2
Adenosquamous carcinoma	9	6.0
Squamous cell carcinoma	4	2.6
Carcinosarcoma	2	1.3
Mucoepidermoid carcinoma	2	1.3
Total	151	100.0

**Figure 1:**
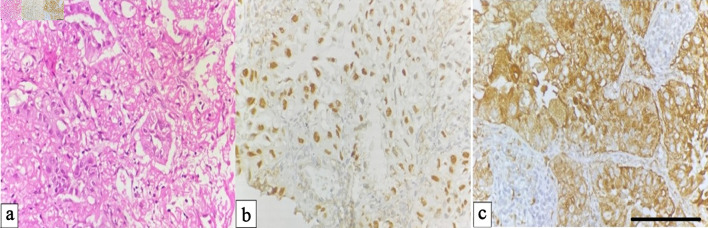
Adenocarcinoma of the lung. (a) Hematoxylin and eosin (H&E) section showing malignant gland-forming epithelial cells (× 400). (b) Immunohistochemical staining demonstrating nuclear positivity for TTF-1 in tumour cells (× 400). (c) Cytoplasmic staining for napsin A confirming adenocarcinomatous differentiation in tumor cells (× 400, scale bar = 50 µm). TTF-1: thyroid transcription factor-1.

Histopathological examination of adenocarcinoma revealed malignant epithelial cells arranged in glandular and papillary configurations with occasional solid nests. Tumor cells exhibited pleomorphic, hyperchromatic nuclei and moderate cytoplasm. IHC confirmed strong nuclear positivity for TTF-1 and granular cytoplasmic staining for napsin A, supporting glandular differentiation (Fig. 1a–c).

### Immunohistochemical marker expression

A comprehensive IHC panel (TTF-1, napsin A, CK7, CK5/6, P63, synaptophysin, and chromogranin) was used to optimize tumor subclassification (Table 2). To further evaluate the diagnostic performance of the immunohistochemical panel, sensitivity and specificity were calculated for key lineage-specific markers. TTF-1 and napsin A demonstrated high sensitivity for adenocarcinoma (85.2% and 94.3%, respectively), with napsin A showing 100% specificity. For squamous differentiation, CK5/6 exhibited high sensitivity (92.3%) and specificity (98.6%), whereas P63 demonstrated lower sensitivity (61.5%) but retained high specificity (98.6%). Synaptophysin and chromogranin showed excellent performance in neuroendocrine tumors, with sensitivities of 96.1% and 100%, respectively, and both markers demonstrating 100% specificity (Table 3). Key observations included that TTF-1 (85%) and napsin A (94%) were highly sensitive for adenocarcinoma. CK5/6 (100%) and P63 (100%) were specific for squamous differentiation. Synaptophysin (96%) and chromogranin (100%) confirmed neuroendocrine origin. Vimentin, smooth muscle actin (SMA), and S-100 protein (S-100) showed focal positivity in carcinosarcoma, indicating epithelial–mesenchymal transition (Table 4).

**Table 2:** IHC Expression Pattern and Specificity (N = 151)

IHC marker	Adeno (+/−)	Neuroendocrine (+/−)	Adenosquamous (+/−)	Squamous (+/−)	Carcinosarcoma (+/−)	Mucoepidermoid (+/−)	Specificity (%)
TTF-1	75/13 (85/15%)	0/26 (0/100%)	1/8 (12.5/87.5%)	0/4 (0/100%)	2/0 (100/0%)	0/2 (0/100%)	48.5
Napsin A	83/5 (94/6%)	0/26 (0/100%)	0/9 (0/100%)	0/4 (0/100%)	0/2 (0/100%)	0/2 (0/100%)	80.0
CK7	80/8 (91/9%)	8/18 (31/69%)	9/0 (100/0%)	4/0 (100/0%)	0/2 (0/100%)	2/0 (100/0%)	25.0
CK5/6	0/88 (0/100%)	0/26 (0/100%)	8/1 (89/11%)	4/0 (100/0%)	0/2 (0/100%)	2/0 (100/0%)	88.6
P63	0/88 (0/100%)	0/26 (0/100%)	4/5 (44/56%)	4/0 (100/0%)	0/2 (0/100%)	2/0 (100/0%)	96.0
Synaptophysin	0/88 (0/100%)	25/1 (96/4%)	0/9 (0/100%)	0/4 (0/100%)	0/2 (0/100%)	0/2 (0/100%)	97.4
Chromogranin	0/88 (0/100%)	26/0 (100/0%)	0/9 (0/100%)	0/4 (0/100%)	0/2 (0/100%)	0/2 (0/100%)	98.5
Vimentin	—	—	—	—	2/0 (100/0%)	—	—
SMA	—	—	—	—	2/0 (100/0%)	—	—
S-100	—	—	—	—	2/0 (100/0%)	—	—
Pan-CK	—	—	—	—	2/0 (100/0%)	—	—

IHC: immunohistochemistry; Adeno: adenocarcinoma; TTF-1: thyroid transcription factor-1; SMA: smooth muscle actin; S-100: S-100 protein; Pan-CK: pan-cytokeratin.

**Table 3:** Diagnostic Performance of Key Immunohistochemical Markers

Marker	Target	Sensitivity	Specificity
TTF-1	Adenocarcinoma	85.2%	95.2%
Napsin A	Adenocarcinoma	94.3%	100%
CK5/6	Squamous	92.3%	98.6%
P63	Squamous	61.5%	98.6%
Synaptophysin	Neuroendocrine	96.1%	100%
Chromogranin	Neuroendocrine	100%	100%

TTF-1: thyroid transcription factor-1;

**Table 4:** IHC Profiles of Metastatic Lesions to the Lung

IHC marker	Breast (n = 10)	Oral (n = 4)	Kidney (n = 6)
ER	7/10	0/4	0/6
PR	7/10	0/4	0/6
HER2	5/10	0/4	0/6
E-cadherin	8/10	0/4	0/6
PAX-2	0/10	0/4	6/6
PAX-8	0/10	0/4	6/6
CD10	0/10	0/4	5/6
CK5/6	0/10	4/4	0/6
P63	0/10	4/4	0/6
CK7	8/10	0/4	0/6
TTF-1	0/10	0/4	0/6

IHC: immunohistochemistry; ER: estrogen receptor; PR: progesterone receptor; HER2: human epidermal growth factor receptor 2; CK: cytokeratin; TTF-1: thyroid transcription factor-1.

### Metastatic lesions

**Figure 2:**
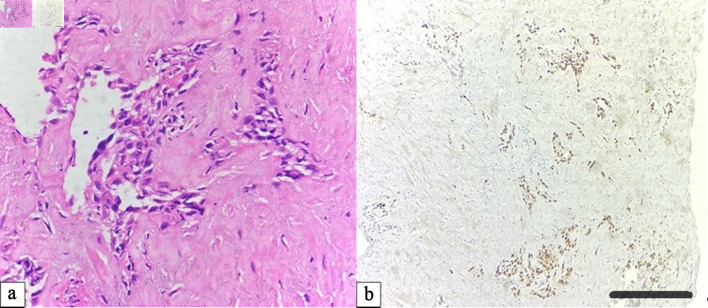
Pulmonary metastasis from oral squamous cell carcinoma. (a) Hematoxylin and eosin (H&E) section showing nests of keratinizing squamous cells with marked pleomorphism and intercellular bridges (× 400). (b) Tumor cells exhibit diffuse nuclear immunoreactivity for P63, supporting squamous origin (× 100, scale bar = 100 µm).

Among the 20 metastatic cases, the lung served as a secondary site for breast carcinoma (10 cases), renal cell carcinoma (six cases), and oral squamous carcinoma (four cases). Breast metastases demonstrated positivity for ER, PR, HER-2, E-cadherin, and CK7. Renal metastases were positive for PAX-2, PAX-8, and CD10. Oral squamous metastases showed strong expression of CK5/6 and P63. All metastatic lesions were TTF-1 negative, confirming their extrapulmonary origin. The oral cavity metastases demonstrated sheets of keratinizing squamous cells with intercellular bridges and strong nuclear P63 positivity, confirming squamous lineage (Fig. 2a, b).

### Rare tumors

**Figure 3:**
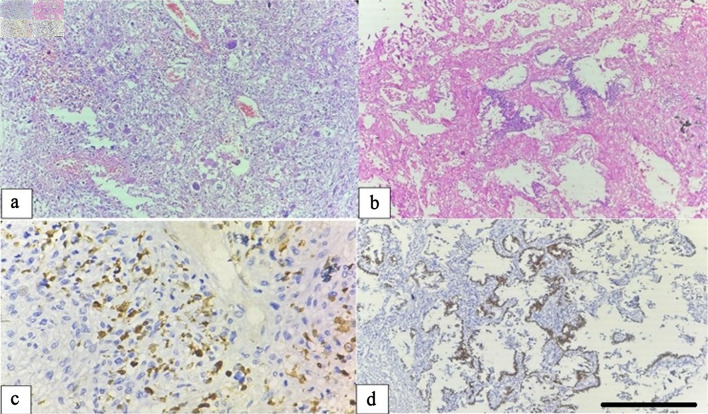
Carcinosarcoma of the lung. (a) Hematoxylin and eosin (H&E) section illustrating the sarcomatous component composed of pleomorphic spindle cells and tumor giant cells with prominent inflammatory infiltration (× 100). (b) Adenocarcinomatous component displaying glandular structures lined by atypical epithelial cells (× 100). (c) The sarcomatous component shows diffuse cytoplasmic positivity for S-100 protein (× 400). (d) The epithelial component exhibits nuclear TTF-1 positivity, confirming biphasic differentiation (× 100, scale bar = 100 µm). TTF-1: thyroid transcription factor-1.

Two cases of carcinosarcoma displayed biphasic morphology with distinct epithelial and mesenchymal components. The adenocarcinoma component showed glandular differentiation and TTF-1 positivity, whereas the sarcomatous component demonstrated tumor giant cells, marked pleomorphism, and positivity for S-100, confirming heterologous mesenchymal differentiation (Fig. 3a–d).

### Correlation analysis between immunomarkers

Correlation analysis revealed strong linear associations between lineage-specific IHC markers (Table 4). TTF-1 vs napsin A in adenocarcinoma showed a strong positive correlation (r = 0.88, P < 0.001), confirming concordant expression in glandular tumors (Table 5). P63 vs CK5/6 in squamous and adenosquamous carcinomas demonstrated an excellent positive correlation (r = 0.93, P < 0.001), underscoring the robustness of this dual-marker panel for squamous differentiation. Synaptophysin vs chromogranin in neuroendocrine neoplasms displayed a highly significant correlation (r = 0.87, P < 0.001), reflecting consistent neuroendocrine lineage expression (Table 5). These correlations reinforce the diagnostic reliability and internal validity of the IHC panel and support its utility in the subclassification of lung tumors when only small tissue biopsies are available.

**Table 5:** Correlation Analysis Between Lineage-Specific Immunohistochemical Markers

Tumor subtype	Immunomarker pair	Correlation coefficient (r)	Significance (P)	Interpretation
Adenocarcinoma	TTF-1 vs napsin A	0.88	< 0.001	Strong positive correlation confirming coexpression of glandular differentiation markers
Squamous/adenosquamous carcinoma	P63 vs CK5/6	0.93	< 0.001	Excellent positive correlation indicating robust concordance of squamous-lineage markers
Neuroendocrine neoplasms	Synaptophysin vs chromogranin	0.87	< 0.001	Highly significant correlation supporting consistent neuroendocrine marker expression

TTF-1: thyroid transcription factor-1; CK: cytokeratin.

## Discussion

This study provides a practice-oriented evaluation of immunohistochemical marker performance in the subclassification of lung tumors, with particular emphasis on real-world diagnostic scenarios involving limited biopsy material. Rather than introducing new biomarkers, our findings focus on validating the reliability, concordance, and clinical applicability of established markers when used as part of an integrated diagnostic panel [18, 23].

This approach aligns with contemporary diagnostic strategies, where IHC plays a central role in resolving morphologically ambiguous cases and guiding tumor classification in accordance with WHO recommendations [15].

The demographic pattern in this study, male predominance (2.1:1) and peak incidence in the fifth to seventh decades, mirrors previously reported epidemiologic distributions in both Asian and Western populations [5, 6]. Tobacco exposure remains a principal determinant, although the rising proportion of adenocarcinoma among non-smokers suggests a multifactorial etiology involving genetic susceptibility, pollution, and occupational hazards [24].

In the era of targeted and immune-based therapies, histopathological classification of lung cancer has become increasingly dependent on IHC profiling. Morphology alone may be insufficient, particularly in small biopsies or poorly differentiated tumors, necessitating marker-based lineage confirmation [16]. The present study underscores that the combination of TTF-1 and napsin A remains the cornerstone for identifying adenocarcinoma, demonstrating high sensitivity and specificity consistent with prior reports [18, 23]. TTF-1, a nuclear transcription factor expressed in alveolar epithelial cells, is indispensable for confirming pulmonary origin, while cytoplasmic napsin A provides complementary confirmation of glandular differentiation [25]. The strong correlation (r = 0.88, P < 0.001) between these markers in our series reinforces their combined diagnostic value and minimizes false negatives, especially when either antigen shows focal loss. The diagnostic performance analysis further supports the reliability of the selected IHC panel. In our study, TTF-1 and napsin A showed high sensitivity for adenocarcinoma, with napsin A achieving complete specificity, reinforcing its role as a robust marker of glandular differentiation. CK5/6 demonstrated excellent sensitivity and specificity for squamous differentiation, while P63, although highly specific, showed comparatively lower sensitivity, highlighting the importance of using combined marker panels. Similarly, synaptophysin and chromogranin exhibited near-perfect diagnostic performance for neuroendocrine tumors. These findings emphasize that a panel-based approach improves diagnostic confidence and minimizes misclassification in small biopsy specimens.

For squamous differentiation, the dual expression of P63 and CK5/6 showed complete concordance (r = 0.93, P < 0.001), in agreement with previous investigations by Kim et al (2013) and Gondha et al (2020) [18, 26]. P63, a p53-homologous transcription factor, identifies basal-type epithelial differentiation, whereas CK5/6 reflects keratinocyte-related cytokeratin expression. Their coexpression provides a highly reliable diagnostic framework for distinguishing squamous carcinoma from adenocarcinoma, particularly in mixed or adenosquamous lesions [26]. Moreover, the use of P40, a more specific ΔNp63 isoform, has been recommended in the latest WHO update, although P63 remains widely adopted in resource-limited settings [16].

In neuroendocrine neoplasms, synaptophysin and chromogranin demonstrated high and concordant positivity (r = 0.87, P < 0.001), confirming their utility as complementary neuroendocrine markers. This observation supports findings by Rudin et al (2021) and Raso et al (2021), emphasizing that dual-marker confirmation enhances diagnostic confidence and helps stratify neuroendocrine tumors ranging from typical carcinoids to small-cell carcinoma [13, 20]. Notably, the expression profile observed in this study mirrors the classical pattern described in large international cohorts, suggesting methodological consistency and biomarker robustness.

A key diagnostic challenge addressed in this study involves differentiating primary lung tumors from secondary metastatic deposits. Among our 20 metastatic cases, the absence of TTF-1 expression proved crucial for excluding primary origin. The integration of organ-specific markers such as ER/PR/HER-2 for breast, PAX-8/PAX-2/CD10 for renal, and CK5/6/P63 for oral cavity metastases provided precise site attribution. Similar marker-based diagnostic strategies have been validated by Vidarsdottir et al (2019) and Carney et al (2015), who emphasized the role of TTF-1 negativity in the recognition of extrapulmonary metastases [21, 22]. These findings underscore the importance of a systematic marker panel in avoiding diagnostic pitfalls, particularly in metastatic settings where treatment decisions hinge on accurate tumor origin. However, CK7 lacks specificity, as it is expressed across a wide range of epithelial malignancies; therefore, it should be interpreted in combination with other lineage-specific markers rather than as a standalone diagnostic indicator.

The correlation analysis conducted in this study provides essential quantitative validation of IHC marker performance. Strong positive correlations across adenocarcinoma, squamous, and neuroendocrine subsets confirmed the internal consistency of the staining protocol and the reproducibility of marker coexpression. This data-driven validation not only reinforces diagnostic accuracy but also supports the development of streamlined minimal marker panels for routine clinical application—particularly valuable in low-resource or high-throughput pathology laboratories [7]. Our findings are comparable to those of Bhatti et al, who reported adenocarcinoma as the predominant histotype (59%), followed by squamous and neuroendocrine carcinomas, and to Ericson Lindquist et al (2021), who demonstrated real-world accuracy exceeding 90% for IHC-based lung cancer diagnosis [6, 7]. The parallel trends in marker specificity—TTF-1/napsin A for adenocarcinoma and CK5/6/P63 for squamous carcinoma—reflect a global consensus in contemporary lung cancer pathology [8].

The integration of histopathology with a targeted IHC panel in this study enhanced diagnostic precision, particularly in small biopsy samples where morphology alone can be inconclusive. However, this study’s limitations include the absence of molecular profiling (e.g., epidermal growth factor receptor (EGFR), anaplastic lymphoma kinase (ALK), Kirsten rat sarcoma viral oncogene homolog (KRAS), or programmed death-ligand 1 (PD-L1)), which is now integral to personalized therapy. Future research incorporating molecular–IHC correlation would provide a more comprehensive diagnostic and prognostic framework [17, 27].

In the current era of precision oncology, molecular profiling has become an essential component of lung cancer diagnosis and management. Alterations in genes such as *EGFR*, *ALK*, and *KRAS*, as well as *PD-L1* expression, are now routinely used to guide targeted and immunotherapy-based treatment strategies [11, 27]. While the present study focuses on immunohistochemical classification, IHC serves as a critical first step in tumor subtyping, thereby directing appropriate molecular testing. The integration of morphological, immunophenotypic, and molecular data provides a comprehensive diagnostic framework aligned with contemporary WHO recommendations [17].

An important strength of this study lies in its data-driven validation of marker concordance and diagnostic performance within a real-world cohort. The strong correlations observed between lineage-specific markers, together with high sensitivity and specificity values, demonstrate the internal consistency and robustness of the applied IHC panel. This is particularly relevant in routine pathology practice, where diagnostic decisions frequently rely on small biopsy specimens and optimized marker selection rather than extensive molecular profiling [10].

By quantitatively supporting marker reliability, our findings provide practical guidance for improving diagnostic confidence and reducing misclassification in daily clinical workflows, consistent with previous real-world studies demonstrating high diagnostic accuracy of IHC-based classification [7, 12].

Overall, this study validates the crucial diagnostic role of IHC in subclassifying lung tumors, differentiating primary from metastatic lesions, and providing lineage-specific insights that align with the WHO 2021 framework. The demonstrated marker correlations confirm the robustness and reproducibility of the applied panel. By integrating morphology with quantitative IHC data, this study supports a precision pathology approach that strengthens diagnostic reliability and informs personalized management strategies for patients with lung cancer.

Taken together, these findings reinforce the role of IHC not merely as a confirmatory tool but as a central, decision-guiding component in lung cancer diagnosis, particularly in biopsy-limited and resource-variable settings.

### Conclusions

In conclusion, this study demonstrates that a focused immunohistochemical panel provides a robust and reproducible approach for lung tumor classification, particularly in small biopsy specimens where morphological assessment alone may be insufficient. The strong concordance observed among lineage-specific markers reinforces their diagnostic reliability, while integrating IHC with histopathology enables accurate distinction between primary and metastatic lesions. These findings highlight the continued relevance of IHC as a practical and clinically impactful tool in lung cancer diagnostics.

## Data Availability

The datasets generated during and/or analyzed during the current study are available from the corresponding authors (MAl) and (AH) on reasonable request.
